# One-Step Assembly of Fluorescence-Based Cyanide Sensors from Inexpensive, Off-The-Shelf Materials

**DOI:** 10.3390/s20164488

**Published:** 2020-08-11

**Authors:** Gregory E. Fernandes, Ya-Wen Chang, Akash Sharma, Sarah Tutt

**Affiliations:** Department of Chemical Engineering, Texas Tech University, Lubbock, TX 79409-3121, USA; ya-wen.chang@ttu.edu (Y.-W.C.); akash.sharma@ttu.edu (A.S.); sarah.tutt@ttu.edu (S.T.)

**Keywords:** fluorescence, non-covalent, complex, sensor, turn-on, off-on, microdiffusion, cyanide, hydrogel, agarose

## Abstract

We report a simple and versatile approach to assemble sensitive and selective fluorescence “turn-on” sensors for cyanide by combining three off-the-shelf materials; namely fluorescent dye, 1-vinyl imidazole polymer, and cupric chloride. The cyanide-sensing species is a non-fluorescent fluorophore-polymer-Cu^2+^ complex; which forms as a result of the imidazole polymer’s ability to bind both fluorophore and fluorescence quencher (Cu^2+^). Cyanide removes Cu^2+^ from these complexes; thereby “turning-on” sensor fluorescence. These sensors are water-soluble and have a detection limit of ~2.5 μM (CN^−^) in water. Our ternary complex-based sensing approach also enables facile emission tuning; we demonstrate the convenient, synthesis-free preparation of blue and green-emitting sensors using distyrylbiphenyl and fluorescein fluorophores, respectively. Furthermore; these ternary complexes are easily immobilized using agarose to create cyanide-sensing hydrogels; which are then used in a simple; novel microdiffusion apparatus to achieve interference-free cyanide analysis of aqueous media. The present study provides an inexpensive approach for portable; interference-free cyanide detection.

## 1. Introduction

An estimated 1.4 million tons of cyanide is produced annually for use in mining, metal processing, plastic and fiber production, tanning, cosmetics, pest control, pharmaceuticals and photography [1,2]. The transportation and widespread use of this highly toxic chemical poses a significant health risk to human beings. Smoke inhalation is another source of cyanide exposure and ~3000–6000 people are exposed to potentially lethal cyanide levels every year from fire smoke [3,4,5,6,7]. In addition, pathogen-mediated cyanogenesis has an acknowledged role in cystic fibrosis lung disease [8,9,10,11,12]. As a result, methods for rapid and sensitive cyanide detection have attracted much interest [5,13].

Fluorescent sensors are an attractive option for cyanide detection, because they are easy to use and exhibit good sensitivity, specificity and response times. The two most common fluorescence-based cyanide sensors are chemodosimeters and metal-ion-displacement probes [13,14,15]. Chemodosimeters change their optical signal upon reaction with cyanide and are thus irreversible probes [13,16]. In contrast, sensors based on cyanide-mediated displacement of metal ions from ion-chelating fluorophores are reversible, but multistep synthesis is often required to produce the ion-chelating fluorophore [17,18,19,20,21,22,23,24,25], making the replication and implementation of such probes difficult for those without synthesis expertise. Thus, a simple, synthesis-free cyanide sensor construction would not only provide a more cost-effective solution, but also broaden both the range of users and the situations in which monitoring is possible (e.g., portable, on-the-go cyanide sensing in the field, in remote areas and by first responders).

In this paper, we show that cyanide-sensing fluorophore-polymer-quencher (FPQ) complexes are easily prepared by mixing as-received, commercially available, fluorescent dye, dye-binding imidazole polymer and fluorescence quencher (Cu^2+^). A distyrlbiphenyl fluorophore is used to produce a blue-emitting “turn-on” sensor that can detect as little as ~2.5 μM cyanide; well below lethal blood cyanide levels (~40 μM) [2,5,26] and the US Environmental Protection Agency’s (EPA) maximum contaminant levels for cyanide in drinking water (~8 μM) [27]. Furthermore, the detection range of these sensors can be modified easily by controlling the relative proportion of quencher within the FPQ complex. We also introduce a sensor immobilization strategy using a biopolymer gel as well as a passive microdiffusion device assembled from off-the-shelf materials, namely, a glass vial and cotton fabric. In contrast with current passive microdiffusion devices [5,28,29], the proof-of-concept assay developed in this work does not need a special analysis cell and is significantly more portable and accident-proof because it traps cyanide in an immobilized cyanide-sensing gel instead of the traditionally used capture solutions. Finally, we demonstrate the versatility of the FPQ sensor platform by using a different fluorophore, fluorescein, to produce a green-emitting sensor. The sensors and assays reported herein represent new, broadly accessible methodologies for inexpensive cyanide analysis without the need for specialized chemical synthesis or device fabrication.

## 2. Materials and Methods

### 2.1. Materials and Instruments

**Scheme 1:**
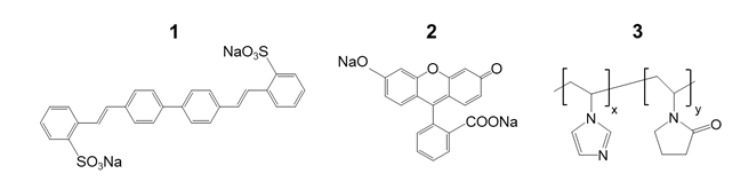
Commercial fluorophores (**1**)–(**2**) and polymer (**3**) used in this work.

All chemicals are commercially available and were used as received. Scheme 1 contains formula representations of all fluorophores and polymers used herein. Tinopal CBS-X **(1)** (88% pure) was purchased from BASF. Pure disodium fluorescein **(2)** was obtained from Acros Organics. A 30 wt% solution of vinylimidazole/vinylpyrrolidone copolymer **(3)** (MW ~70,000 g/mol) was obtained from BASF (Sokalan HP 56K).

CuCl_2_.2H_2_O, NaCN, NaI, NaSCN, NaCl, Na_2_S.9H_2_O, Na_2_CO_3_, NaNO_2_, NaNO_3_, NaHCO_3_, NH_4_OH, CH_3_COOH, C_2_H_3_O_2_Na·3H_2_O, Na_2_SO_3_, Na_2_SO_4_, NaHSO_4_·H_2_O, NaHPO_4_, Na_3_PO_4_·12H_2_O, NaBr, NaF, HNO_3_, H_2_SO_4_, BSA, Ethanol, Acetone, Triton X-100 and HPLC grade Acetonitrile were purchased from Fisher Scientific. Bi(NO_3_)_3_.5H_2_O was purchased from Sigma-Aldrich. pH = 5, MF254, alumina-backed TLC silica plates were purchased from Agela Technologies. Low gelling temperature agarose was purchased from Fisher Scientific. Bleached, brightened cotton was purchased from Testfabrics Inc. Twenty milliliter borosilicate glass scintillation vials were purchased from Fisher Scientific.

Fluorescence spectra were recorded using an Agilent Cary Eclipse fluorescence spectrophotometer, a Horiba Aqualog fluorimeter and a Synergy H1 Hybrid Multi-Mode Reader form BioTek. All emission spectra were recorded with an excitation wavelength of 350 nm for **(1)** and 480 nm for **(2)**. One centimeter pathlength cuvettes were used for fluorimeter readings and Costar 96-well black polystyrene plates were used for fluorescence plate reader studies. FTIR analyses were performed using a Bruker Vertex 70 Hyperion 2000 Platinum ATR. Spectra were recorded at a resolution of 2 cm^−1^ with 32 scans.

### 2.2. Thin Layer Chromatography

**Figure 1:**
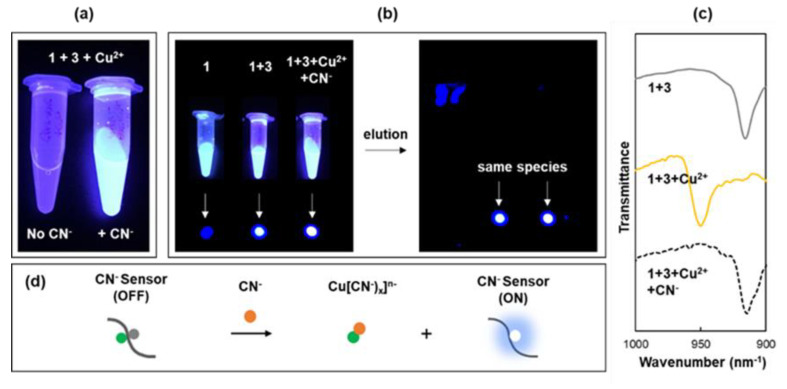
(**a**) Photograph of an aqueous mixture containing 0.8 mM **1**, 0.4 mM **3** and 16 mM Cu^2+^ (left) and an aqueous mixture containing 0.8 mM **1**, 0.4 mM **3**, 16 mM Cu^2+^ and 300 mM CN^−^ (right) under UV illumination. (**b**) UV-illuminated (λ_EX_ = 350 nm) spots of 0.8 mM **1** only, a 0.8 mM **1** + 0.4 mM **3** mixture, and a 0.8 mM **1** + 0.4 mM **3** + 16 mM Cu^2+^ + 300 mM CN^−^ mixture on a TLC plate, before (left) and after (right) elution with solvent. TLC solvent system: acetonitrile/water 25:75 (w/w). TLC plates with standard silica treatment and aluminium backing are used. (**c**) Mid-FTIR spectra in the low frequency region for a **1+3** complex (gray line), and a **1+3+Cu^2+^** complex before (yellow line) and after (dashed black line) treatment with excess CN^−^. The **1+3** complex has 2:1 (**1:3**) stoichiometry. The **1+3+Cu^2+^** complex has 2:1:40 (**1:3:Cu^2+^**) stoichiometry. (**d**) Schematic representation of the effect of CN^−^ addition on **1+3+Cu^2+^** complexes.

Amounts of 0.5 μL of each of the following aqueous solutions were spotted on standard aluminum backed silica thin layer chromatography (TLC) plates and run side-by-side to generate the TLC data shown in Figure 1:

i) 0.8 mM **1** in deionized water (referred to as **1** in Figure 1b).
ii) 0.8 mM **1** + 0.4 mM **3** in deionized water (referred to as **1 + 3** in Figure 1b).
iii) 0.8 mM **1** + 0.4 mM **3** + 16 mM Cu^2+^ + 300 mM CN^−^ in deionized water (referred to as **1+3+Cu^2+^+CN^−^** in Figure 1b).

The solvent system comprised 25 wt% acetonitrile and 75 wt% deionized water.

### 2.3. Attenuated Total Reflectance-Fourier Transform Infrared Studies

Purification of the various complexes described herein followed the procedure of Fernandes et al. [30]. For **1+3** complexes, 3.25 g of a **1+3** mixture containing 0.8 mM **(1)** and 0.4 mM **(3)** was thoroughly mixed and sonicated with 125 g of a solvent system containing 50 g of ethyl acetate and 75 g of acetone, which resulted in selective precipitation of the **1+3** complex. The resulting precipitate was washed, dried, and characterized using ATR-FTIR (referred to as **1+3** in Figure 1c).

For the preparation and purification of **1+3+Cu^2+^** complexes, 3.25 g of a **1+3** mixture containing 0.8 mM **(1)** and 0.4 mM **(3)** was thoroughly mixed and sonicated with 1 mL of 0.5 M Cu^2+^ (aqueous) to saturate the **1+3** complex with Cu^2+^ ions. The resulting precipitate was washed, dried and characterized using ATR-FTIR (referred to as **1+3+Cu^2+^** in Figure 1c).

To prepare CN^−^-treated **1+3+Cu^2+^** samples, washed **1+3+Cu^2+^** precipitate was prepared by repeating the procedure above, dissolved in 1 mL of 0.5 M CN^−^ (aqueous), dried and characterized using ATR-FTIR (referred to as **1+3+Cu^2+^+CN^−^** in Figure 1c).

## 3. Results and Discussion

### 3.1. Sensing Mechanism

Our sensor is a quenched, non-fluorescent, aqueous mixture comprising fluorophore **(1)**, 1-vinyl imidazole polymer (**3**) and Cu^2+^ in 2:1:40 stoichiometry (Figure 1a, left). Our recent work [30] demonstrates that the observed fluorescence quenching is caused by the formation of **1+3+Cu^2+^** complexes; a direct result of the imidazole polymer’s ability to bind both fluorophore and fluorescence quencher (Cu^2+^). Our initial choice of 2:1:40 (**1**:**3**:**Cu^2+^**) stoichiometry is to minimize the amount of unbound fluorophore and at the same time, to saturate the complex with Cu^2+^ [30], resulting in a fully quenched solution with minimal background fluorescence (Figure 1a, left). Upon the addition of cyanide to **1+3+Cu^2+^** complexes, we observe a significant increase in solution fluorescence (Figure 1a, right). Since CN^−^ associates strongly with Cu^2+^ [31], a known fluorescence quencher [32,33], we suspect that the observed fluorescence recovery is caused by cyanide-induced removal or the displacement of Cu^2+^ from the **1+3+Cu^2+^** complex.

To confirm that cyanide removes Cu^2+^ from **1+3+Cu^2+^** complexes, we run chromatograms for fluorophore **(1)** only, **1+3** mixtures and CN^−^-treated **1+3+Cu^2+^** mixtures side-by-side on the same TLC plate (Figure 1b). As reported previously [30], retention times for fluorophore **(1)** only and **1+3** mixtures differ significantly on account of spontaneous fluorophore-polymer complexation in **1+3** mixtures. Here, we show that retention times for **1+3** mixtures match those of CN^−^-treated **1+3+Cu^2+^** mixtures, which strongly suggests that high doses of CN^−^ can remove all bound copper from **1+3+Cu^2+^** complexes. The TLC data also show that CN^−^ treatment does not displace fluorophore from the **1+3+Cu^2+^** complex, as no fluorescent spot at retention time corresponding to free fluorophore **(1)** is observed for CN^−^-treated **1+3+Cu^2+^** samples. Additional evidence for CN^−^-mediated removal of bound copper from **1+3+Cu^2+^** complexes comes from ATR-FTIR, where the effect of CN^−^ exposure on the coordinate bonds between Cu^2+^ and the imidazole ligands present in **1+3** complexes is monitored (Figure 1c).

Mid-FTIR spectra (Figure 1c) show that exposure of **1+3** complexes to Cu^2+^ results in the disappearance of the band at 915 cm^−1^ and the occurrence of a band at 950 cm^−1^. For 1-vinyl imidazole polymers, this is a signature of replacement of imidazole-H_2_O-imidazole bridged water with Cu^2+^ to from imidazole-Cu^2+^-imidazole complexes [34,35,36]. The Mid-FTIR spectra for CN^−^-treated **1+3+Cu^2+^** complexes shows a reverse effect, namely, the disappearance of the 950 cm^−1^ band and the recovery of the band at 915 cm^−1^. This shows that CN^−^ treatment replaces the imidazole-Cu^2+^-imidazole coordinate bonds within the **1+3+Cu^2+^** complexes with imidazole-H_2_O-imidazole bonds, thus proving the cyanide-mediated displacement of Cu^2+^ from **1+3+Cu^2+^** complexes.

Taken together, the TLC and ATR-FTIR findings show that the **1+3+Cu^2+^** complex functions as a Cu^2+^-displacement-based fluorescence “turn-on” probe for cyanide (Figure 1d). Unlike other Cu^2+^-displacement-based cyanide probes [17,18,19,20,21,22,23,24,25], our sensor is assembled by one-step mixing of as-received commercial materials and is thus “synthesis-free”. The cyanide sensor described in this paper would allow researchers to quickly assemble inexpensive cyanide probes without the need for any synthesis-related training, expertise, or equipment. We refer to the **1+3+Cu^2+^** mixture with 2:1:40 stoichiometry as sensor **A**.

### 3.2. Sensing Performance and Sensitivity Optimization

**Figure 2:**
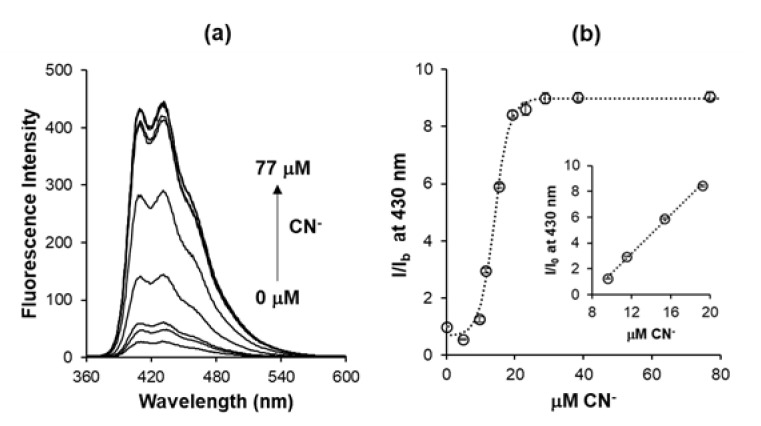
(**a**) Fluorescence emission spectra of 0.1 μM sensor **A** (a 0.2 μM **1 +** 0.1 μM **3** + 4 μM Cu^2+^ mixture) upon addition of increasing concentrations of CN^−^ (0–77 μM) in aqueous solution. (**b**) Fluorescence recovery (I/I_b_) of 0.1 μM sensor **A** (open circles) upon addition of CN^−^. The dashed line is a sigmoidal fit and is intended to guide the eye. The insert is the recovery plot in the linear dose response region. For all studies reported in Figure 2, error bars represent three standard deviations, the solvent used is 0.5wt% Triton X-100 in deionized water, and λ_EX_ = 350nm. Sensor **A** is a **1+3+Cu^2+^** complex with 2:1:40 (**1:3:Cu^2+^**) stoichiometry.

Emission spectra of sensor **A**, recorded before and after CN^−^ addition, show an increase in emission intensity with increasing CN^−^ concentration, thus confirming that sensor **A** acts as a fluorescence “turn-on” probe for CN^−^ (Figure 2a). Fluorescence recovery (I/I_b_) for sensor **A**, where I is sensor **A**’s peak emission intensity in the presence of CN^−^ and I_b_ is the background fluorescence from sensor **A** in the absence of CN^−^, is linear in the 10-20 μM CN^−^ range and is complete upon exposure to ~40 μM CN^−^ (Figure 2b). The detection limit of sensor A is therefore ~10 μM, well below the lethal blood cyanide levels in smoke inhalation victims (~40 μM) [2,5,26], making sensor **A** potentially useful for the detection of harmful levels of CN^−^ in the blood of smoke inhalation victims and firefighters.

**Figure 3:**
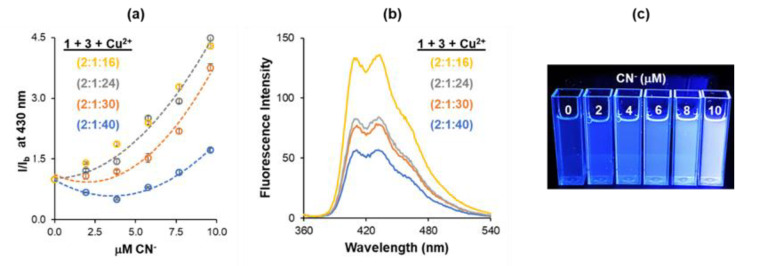
(**a**) Fluorescence recovery (I/I_b_) of aqueous mixtures containing 0.2 μM **1** + 0.1 μM **3** + X μM Cu^2+^ upon addition of increasing concentrations of CN^−^ (0–10 μM); X = 1.6 μM (yellow circles), 2.4 μM (gray circles), 3 μM (orange circles) and 4 μM (blue circles) Cu^2+^ respectively; dashed lines are guides to the eye. (**b**) Fluorescence emission spectra of aqueous mixtures containing 0.2 μM **1** + 0.1 μM **3** + X μM Cu^2+^; X = 1.6 μM (yellow curve), 2.4 μM (gray curve), 3 μM (orange curve) and 4 μM (blue curve) Cu^2+^ respectively. (**c**) Photograph of aqueous mixtures containing 0.2 μM **1** + 0.1 μM **3** + 2.4 μM Cu^2+^ as a function of increasing CN^−^ levels; taken under UV illumination (λ_EX_ = 350 nm). For all studies reported in Figure 3, error bars represent three standard deviations, the solvent used is 0.5wt% Triton X-100 in deionized water, and λ_EX_ = 350 nm.

Efforts to improve sensor **A**’s performance involve adjusting the formulation of the **1+3+Cu^2+^** complex, as summarized in Figure 3a. We observe that (i) the sensor’s detection limit can be substantially improved by reducing the relative proportion of quencher (Cu^2+^) in the **1+3+Cu^2+^** mixture, and (ii) 2:1:24 (**1:3:Cu^2+^**) stoichiometry represents the limit beyond which no additional sensitivity is gained by lowering relative Cu^2+^ levels. As expected, these sensitivity improvements come at the cost of compromised background fluorescence, but the effect is minimal for mixtures with 2:1:24 (**1:3:Cu^2+^**) stoichiometry and only becomes pronounced in 2:1:16 (**1:3:Cu^2+^**) mixtures, where we see a two-fold increase (Figure 3b).

We therefore find 2:1:24 (**1:3:Cu^2+^**) stoichiometry to be optimal; this ternary mixture remains non-fluorescent or “off” in the absence of cyanide (Figure 3b), but (i) enables the quantitation of <10 μM CN^−^ with an analytical detection limit of ~2.5 μM CN^−^ (See Supplementary Information) and (ii) exhibits a visually discernable fluorescence recovery at CN^−^ levels as low as 5 μM (Figure 3c). Since the EPA has set the maximum contaminant level for cyanide in drinking water at ~8 μM [27], our optimized sensor could potentially serve as a candidate for the fluorogenic, naked-eye detection of harmful levels of CN^−^ in drinking water.

**Figure 4:**
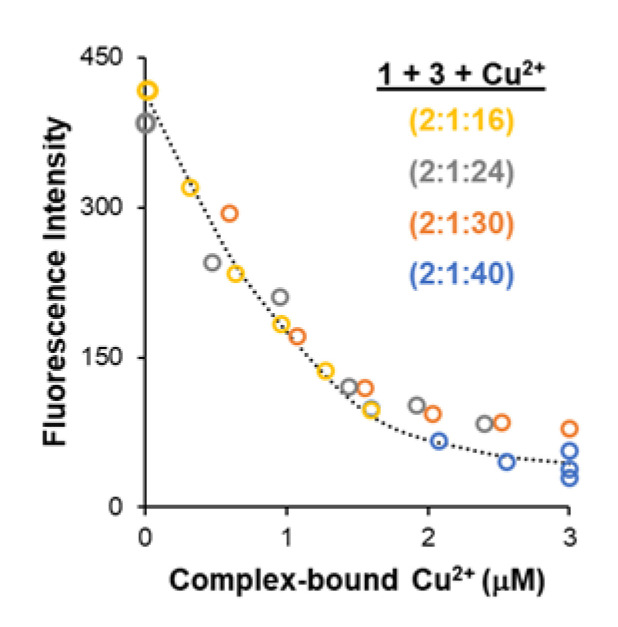
Superposition of CN^−^ dose response curves from Figure 3a, obtained by plotting fluorescence (λ_EX_ = 350 nm; λ_EM_ = 430 nm) as function of bound Cu^2+^ within the ternary (**1+3+Cu^2+^**) complex; the dotted line represents the expected fluorescence response upon Cu^2+^ removal from the ternary complexes. We assume that CN^−^-induced Cu^2+^ displacement from **1+3+Cu^2+^** complexes involves formation of [Cu(CN)_x_]^1−x^ complexes; x = 4 (blue, orange and gray circles) and 6 (yellow circles).

The tunability of the CN^−^ detection range and limit can be understood by considering the fluorescence intensity as a function of bound Cu^2+^ within the ternary complex. In the absence of CN^−^, fluorescence quenching by Cu^2+^ is described by a Stern–Volmer plot, I/I_0_ = (1 + K_sv_ [Cu^2+^])^−1^, where I_0_ and I are the peak fluorescence intensities for **1+3** mixtures before and after addition of Cu^2+^ (quenching constant, K_sv_ ~1.68 μM^−1^; see Supplementary Information). Thus, the expected fluorescence recovery upon Cu^2+^ removal from **1+3+Cu^2+^** complexes can be described by this very curve, as represented by the fluorescence intensity as a function of **1+3**-bound Cu^2+^ levels (Figure 4, dotted line). From our previous work [30], we know that each **1+3** complex binds ~30 Cu^2+^ ions. Therefore, by assuming that (i) free Cu^2+^ forms [Cu(CN)_4_]^3−^ complexes [18] and (ii) CN^−^-induced Cu^2+^ displacement from **1+3+Cu^2+^** complexes involves formation of [Cu(CN)_x_]^1−x^ (x = 4~6) complexes, superposition of all dose-response curves shown in Figure 3a onto a single master-curve is achieved (Figure 4). This superposition is further confirmation that **1+3+Cu^2+^** complexes function as Cu^2+^-displacement-based fluorescence “turn-on” probes for cyanide.

### 3.3. Overcoming Interference

**Figure 5:**
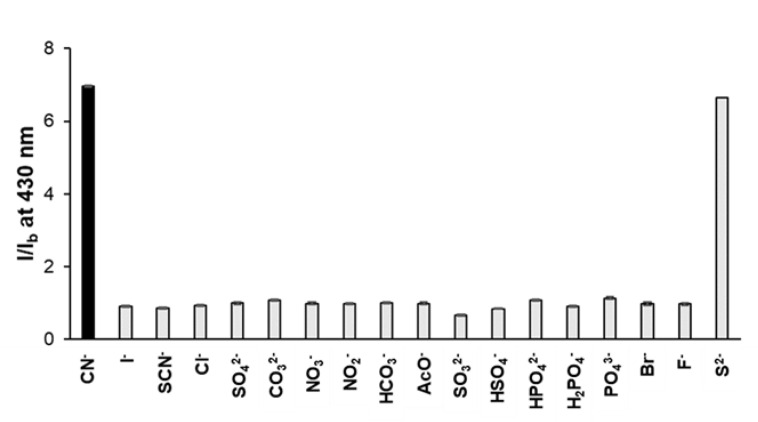
Fluorescence recovery (I/I_b_) for 0.1 μM sensor **A** (a 0.2 μM **1** + 0.1 μM **3** + 4 μM Cu^2+^ mixture) in the presence of 25 μM of various anions; the solvent used is 0.5wt% Triton X-100 in deionized water and λ_EX_ = 350 nm.

**Figure 6:**
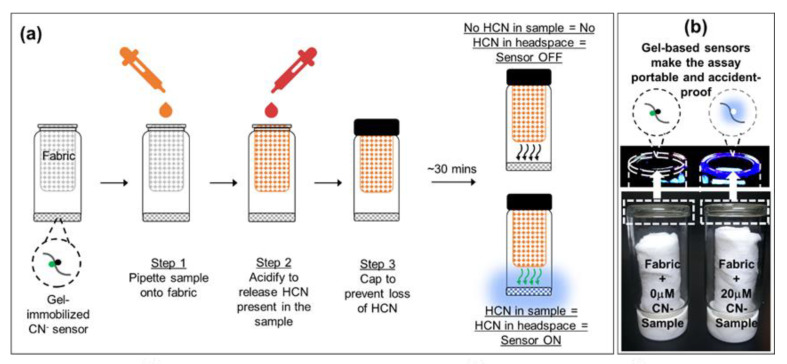
(**a**) Schematic illustration of the microdiffusion assay introduced in this work for separating cyanide from interfering species present withing the sample. (**b**) Photographs of inverted assay vials demonstrating how sensor immobilization within a gel makes the assay resistant to disturbances. The acidification reagent comprises 1g/L Bi(NO_3_)_3_.5H_2_O in 0.55M H_2_SO_4_. All pictures are taken 30 min after acidification under UV illumination (λ_EX_ = 350 nm). The gel-immobilized sensor comprises 0.5% agar, 0.26 μM fluorophore, 0.75 μM polymer and 2 μM Cu^2^ in DI water.

Figure 5 depicts the fluorescence recovery (I/I_b_) of sensor **A** upon addition of various anions and shows that species with high Cu^2+^ affinity (e.g., S^2−^) [37,38] cause significant interference. Since complex biological fluids such as wound exudate, sputum and blood contain chemical interferents such as H_2_S [39,40,41], cytokines [42] and bacterial secretions [43] and cause optical interference due to their color and opacity [42], practical implementation of sensor **A** requires isolation of cyanide from sources of interference. Herein, we introduce a simple microdiffusion device that effectively separates cyanide from non-volatile interferents and enables relatively rapid (~30 min), interference-free detection of cyanide using two inexpensive, off-the-shelf materials, namely, a glass vial and a cotton fabric (Figure 6).

**Figure 7:**
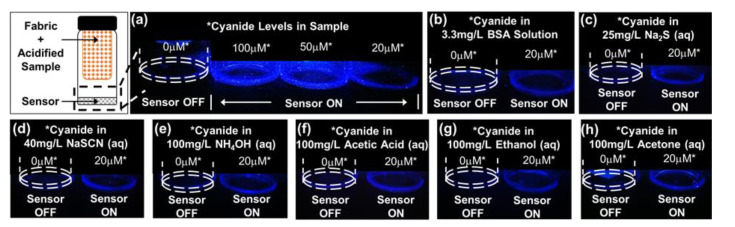
(**a**) Photographs, taken under UV illumination, show that the sensor, located at the bottom of the assay vial, while never in contact with the sample, produces a visually discernible response for samples containing ≥20 μM cyanide. (**b**) Photographs, taken under UV illumination, show that the assay overcomes interference from non-volatile species in the sample as illustrated using BSA solutions. (**c**)–(**h**) Photographs, taken under UV illumination, show that inclusion of bismuth nitrate in the acidification reagent overcomes sulfide interference and does not produce false positives with other common volatile species expected in biological samples. The acidification reagent comprises 1g/L Bi(NO_3_)_3_.5H_2_O in 0.55 M H_2_SO_4_. All pictures are taken 30 min after acidification under UV illumination (λ_EX_ = 350 nm). The gel-immobilized sensor used comprises 0.5% agar, 0.26 μM fluorophore, 0.75 μM polymer and 2 μM Cu^2^ in DI water.

To assemble the microdiffusion device, a warm (50 °C) sensor **A** solution containing 0.5 wt% dissolved agar is pipetted into the bottom of a vial which, upon-cooling, produces a cyanide-sensing hydrogel. These gel-based sensors stay affixed to the bottom of the vial even when the vial is inverted (Figure 6b), thus making the device portable and accident-proof. Next, (i) the mouth of the vial is plugged with cotton fabric, (ii) the fabric is sequentially wet with sample and acid, which converts non-volatile CN^−^ present in the sample into volatile HCN and (iii) the vial is capped to prevent loss of HCN. Cyanide released into the vial headspace eventually dissolves in the hydrogel sensor located at the bottom of the device and “turns-on” fluorescence. This simple, qualitative assay can detect as little as 20 μM CN^−^ in 3 mL of sample (Figure 7a).

The fabric not only absorbs the sample and isolates it from the sensor, but also promotes evaporation of volatile compounds contained within the sample. In doing so, the fabric separates cyanide from non-volatile interferents such as proteins, siderophores and colored species present in the sample. Validation of this approach is provided in Figure 7b, where the assay is used to achieve interference-free cyanide analysis of a solution of serum albumin, a common, non-volatile, Cu^2+^-binding interferent found in blood (HSA level in blood ~3.3 mg/mL).

A number of volatile compounds including organic acids, thiocyanic acid, alcohols, ketones, sulfides, and ammonia are expected to be released into the headspace of the apparatus shown in Figure 6 upon the acidification of biological matrices such as wound exudate, sputum or blood [5,44,45,46,47,48]. H_2_S is of particular concern because of the high affinity of sulfide^19^ for Cu^2+^ (Figure 5) [14,49,50,51]. We show that sulfide interference is removed by dissolving bismuth nitrate in the acidification reagent (Figure 7c); bismuth reacts with sulfide contained in the sample to form insoluble bismuth sulfide [52]. Our preliminary tests using other representative volatile species (Figure 7d–h) at levels expected in biological matrices, show no evidence of interference and suggest that the simple microdiffusion assay introduced in this work could be developed into a portable device for interference-free cyanide detection in drinking water, wound exudate, sputum and blood.

The microdiffusion device described in this work shares a feature common to all passive microdiffusion devices, namely, design simplicity. It also provides analysis times comparable to the fastest passive microdiffusion-based assays [5,28,29], However, unlike the classical Conway passive microdiffusion device and its variants, (i) our apparatus does not require a special analysis cell and is assembled using just fabric and a glass vial, and (ii) our device captures cyanide in an immobilized cyanide-sensing gel instead of a capture solution and is therefore more resistant to disturbances; making it more portable and accident-proof. On the whole, the proof-of-concept assay introduced herein represents a valuable new technique for rapid, interference-free, field-portable cyanide detection.

### 3.4. Cyanide Sensing Hydrogels

**Figure 8:**
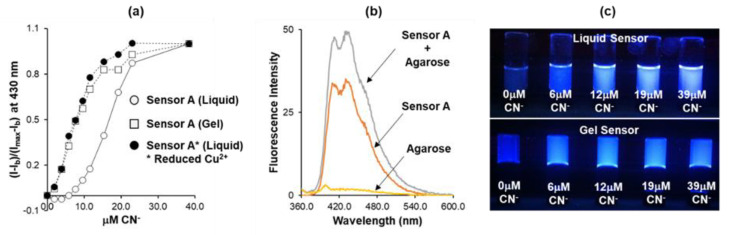
(**a**) Normalized fluorescence recovery of a 0.2 μM **1** + 0.1 μM **3** + 4 μM Cu^2+^ liquid mixture (or 0.1 μM sensor **A-liquid**) (open circles), a 0.2 μM **1** + 0.1 μM **3** + 4 μM Cu^2+^ + 0.5 wt% agarose gel mixture (or 0.1 μM sensor **A-gel**) (open squares) and a 0.2 μM **1** + 0.1 μM **3** + 1.6 μM Cu^2+^ liquid mixture (or 0.1 μM sensor **A**-**reduced Cu^2+^**-**liquid**) (filled circles), upon addition of CN^−^ (λ_EX_ = 350 nm). (**b**) Fluorescence emission spectra (λ_EX_ = 350 nm; T = 40 °C) of aqueous mixtures containing 0.5 wt% agarose only (yellow curve), 0.2 μM **1** + 0.1 μM **3** + 4 μM Cu^2+^ (or 0.1 μM sensor **A**) only (orange curve), and an aqueous mixture containing 0.1 μM sensor **A** and 0.5 wt% agarose (grey curve). (**c**) Photographs of CN^−^-induced fluorescence response of liquid sensor **A**-**reduced Cu^2+^** or sensor A* (top) and gel-immobilized sensor **A** (bottom) taken under UV illumination (λ_EX_ = 350 nm); vial inversion demonstrates gel-immobilization.

Figure 8a compares the cyanide-induced fluorescence recovery of sensor **A** in solution (open circles) with that of sensor **A** within an agarose hydrogel (open squares) and shows that gel immobilization increases sensor **A**’s cyanide sensitivity. As discussed earlier (Figure 3a), the cyanide sensitivity of **1+3+Cu^2+^** complexes like sensor **A** can be improved by lowering relative Cu^2+^ levels within the complex. Since D-galactose, a component of the agarobiose repeat unit that makes up agarose, is known to coordinate with Cu(II) [53,54,55,56,57], we hypothesize that agarose displaces a fraction of the bound Cu^2+^ present within sensor **A**, thereby decreasing the relative Cu^2+^ level within the **1+3+Cu^2+^** complex, which improves its sensitivity to lower doses of cyanide.

To confirm that agarose removes Cu^2+^ from **1+3+Cu^2+^** complexes, we record fluorescence emission spectra of agarose only, sensor **A** (a **1+3+Cu^2+^** complex) only, and a mixture of agarose and sensor **A** (Figure 8b). Addition of non-fluorescent agarose to sensor **A** results in a small but measurable increase in solution fluorescence which supports the hypothesis that agarose removes a fraction of the fluorescence quencher (Cu^2+^) from sensor **A**. It is unlikely that agarose removes the anionic fluorophore (**1**) from **1+3+Cu^2+^** complexes, since the active dye-binding groups on agarose, namely, the pendant hydroxyl groups on D-galactose [58,59], are ineffective at binding anionic dyes; a reason why agarose has been investigated as an absorbent for cationic dyes [59,60,61,62], but is not used for removal of anionic dyes from water.

Titration studies (Figure 8a) reveal that the cyanide dose–response curve for a solution phase **1+3+Cu^2+^** complex with 2:1:16 (**1:3:Cu^2+^**) stoichiometry (filled circles) matches that of agarose-immobilized **1+3+Cu^2+^** complex with 2:1:40 (**1:3:Cu^2+^**) stoichiometry (sensor **A**; open squares). This means that when 0.1 μM sensor **A** (a 0.2 μM **1** + 0.1 μM **3** + 4 μM Cu^2+^ mixture) is immobilized inside 50 μM of agarose, the agarose removes ~2.4 μM Cu^2+^ from the sensor, implying that twenty agarose molecules are required to displace one Cu^2+^ ion from the Cu^2+^-chelating imidazole groups present in sensor **A**. This result is supported by studies documenting the relatively poor Cu^2+^ complexation properties of carbohydrates such as D-galactose [53,55,56,57] in comparison with other biological ligands such as imidazole [35,36,63,64,65]. It is this relative inertness of agarose to Cu^2+^, however, that makes it an excellent candidate for producing cyanide sensing hydrogels (Figure 8c) without destroying the sensor (a **1+3+Cu^2+^** complex).

### 3.5. Emission Tuning

**Figure 9:**
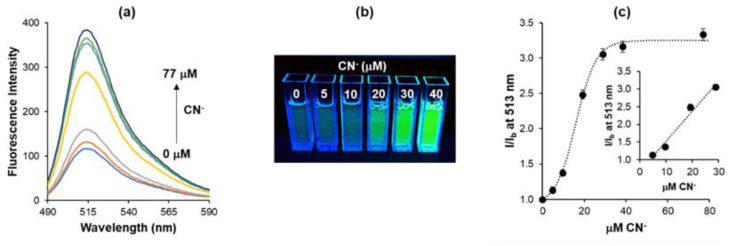
(**a**) Fluorescence emission spectra of a 0.2 μM **2** + 0.1 μM **3** + 16 μM Cu^2+^ mixture upon addition of increasing concentrations of CN^−^ (0–77 μM) in aqueous solution. (**b**) Photograph of aqueous mixtures containing 0.2 μM **1** + 0.1 μM **3** + 16 μM Cu^2+^ as a function of increasing CN^−^ levels. (**c**) Fluorescence recovery (I/I_b_) of a 0.2 μM **1** + 0.1 μM **3** + 16 μM Cu^2+^ mixture upon addition of CN^−^. For all studies reported in Figure 5, error bars represent three standard deviations, the solvent used is 0.5wt% Triton X-100 in deionized water, and λ_EX_ = 480 nm.

Modifying sensor emission involves simply replacing the fluorophore component of the ternary fluorophore-polymer-quencher complex. To transform sensor **A** into a green-emitter, we replace the distyrylbiphenyl fluorophore **(1)** with green-emitting disodium fluorescein **(2)**. Figure 9 confirms that **2+3+Cu^2+^** mixtures function as green-emitting, “turn-on” fluorescence probes for CN^−^. Furthermore, both blue (**1+3+Cu^2+^**) and green (**2+3+Cu^2+^**) complexes show similar cyanide sensitivity and linear dose-response ranges (Figure 9b,c). Thus, in addition to simplifying sensor construction, the approach described in this paper also enables facile tuning of sensor emission colour without compromising cyanide sensitivity.

## 4. Conclusions

We combined three inexpensive commercial materials—namely fluorescent dye, 1-vinyl imidazole polymer and cupric chloride—to produce sensitive and selective “off-on” fluorescence sensors for CN^−^. The key cyanide-sensing species is a non-fluorescent fluorophore-polymer-Cu^2+^ complex, which forms as a result of the imidazole polymer’s ability to bind both fluorophore and fluorescence quencher (Cu^2+^). CN^−^ removes or displaces Cu^2+^ from these complexes, thus “switching-on” sensor fluorescence. Our optimized sensors have analytical (2.5 μM) as well as naked eye (5 μM) detection limits that make them good candidates for the cyanide monitoring of drinking water as well as detection of harmful cyanide levels in the blood of smoke inhalation victims. Unlike other Cu^2+^ displacement-based cyanide probes, our sensor is assembled by one-step mixing of as-received commercial materials and is thus “synthesis-free”. Our approach also enables the facile tuning of sensor emission color without compromising cyanide sensitivity. Finally, we used a glass vial, fabric and an agarose-immobilized sensor to assemble a simple, inexpensive, off-the-shelf microdiffusion apparatus that provides interference-free, qualitative cyanide analysis (20 μM detection limit). Unlike other microdiffusion devices, our apparatus does not require a special analysis cell and is more portable and accident-proof because it captures cyanide in an immobilized, disturbance-resistant CN^−^-sensing gel instead of a capture solution. The cyanide sensors, formulations and microdiffusion apparatus described in this paper would allow researchers to quickly assemble inexpensive cyanide assays without the need for any synthesis-related training, expertise or equipment.

## Supplementary Materials

The following are available online at https://www.mdpi.com/1424-8220/20/16/4488/s1, Figure S1: Estimating the quenching constant of **1** + **3** + Cu^2+^ complexes, Figure S2: Analytical detection limit of optimized, CN^−^ sensing, **1** + **3** + Cu^2+^ complex.
