# Supplementary material for: One-Step Assembly of Fluorescence-Based Cyanide Sensors from Inexpensive, Off-The-Shelf Materials
Source: Sensors (Basel). 2020 Aug 11;20(16):4488. doi: 10.3390/s20164488 (PMC7472291; doi:10.3390/s20164488)
Supplement: Supplementary file 1 [file sensors-20-04488-s001.pdf]

Supplementary Material

One-Step Assembly of Fluorescence-Based Cyanide Sensors from Inexpensive, Off-The-Shelf Materials

Gregory E. Fernandes *, Ya-Wen Chang, Akash Sharma and Sarah Tutt

Department of Chemical Engineering, Texas Tech University, Lubbock, TX 79409-3121, USA; ya-wen.chang@ttu.edu (Y.-W.C.); akash.sharma@ttu.edu (A.S.); sarah.tutt@ttu.edu (S.T.)

* Correspondence: gregory.fernandes@ttu.edu

Estimating the quenching constant of 1 + 3 + Cu^{2+} complexes

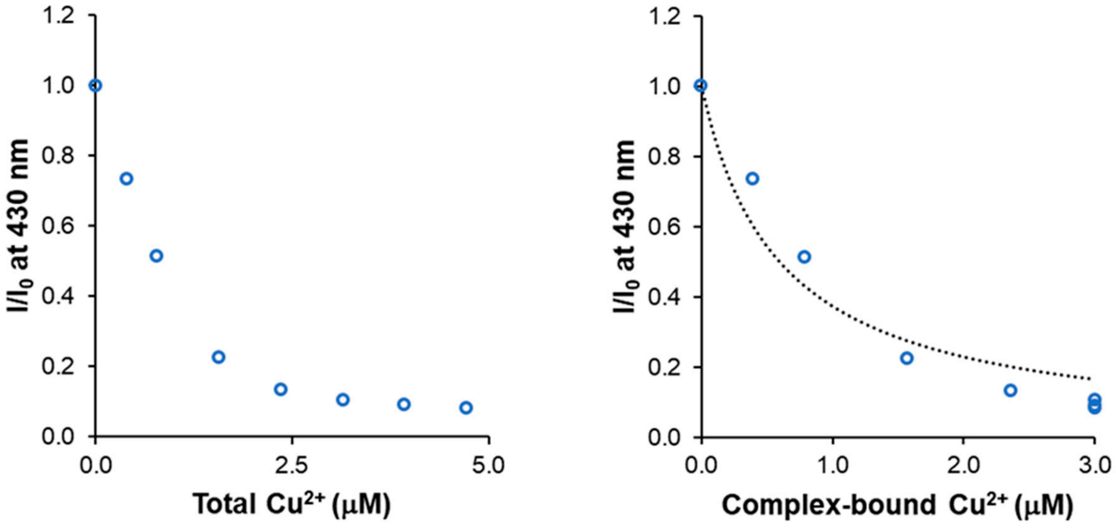

(**a**) (**b**)

Figure S1. (**a**) Normalized fluorescence response (I/I_0) of a 0.2 mM **1** + 0.1 mM **3** mixture as a function of increasing Cu^{2+} levels. Excitation wavelength = 350nm. (**b**) Normalized fluorescence response (I/I_0) of a 0.2 mM **1** + 0.1 mM **3** mixture as a function of Cu^{2+} bound within the **1** + **3** complex. Excitation wavelength = 350nm. The dashed curve is the best fit to the Stern–Volmer equation ($I/I_0 = (1+K_{sv}\,[Cu^{2+}])^{-1}$).

To estimate the quenching constant (K_{sv}) for **1** + **3** + Cu^{2+} complexes, we collect dose response data for **1** + **3** mixtures in the presence of increasing Cu^{2+} levels (Figure S1a). Next, we transform the x-axis from "Total Cu^{2+}" to "Complex-bound Cu^{2+}" (Figure S1b). We can do this because, in previous studies, we have shown that each **1** + **3** complex is able to bind a maximum of 30 Cu^{2+} ions [1], a number that is directly corroborated by the data in Figure S1, which clearly shows that

Cu^{2+} levels > 3 mM cause no additional quenching in 0.1 mM **1 + 3** mixtures. Finally, we fit the data in Figure S1 (**b**) to the Stern–Volmer equation to obtain the K_{sv} ~ 1.68 mM.

Analytical detection limit of optimized, CN^- sensing, 1 + 3 + Cu^{2+} complex

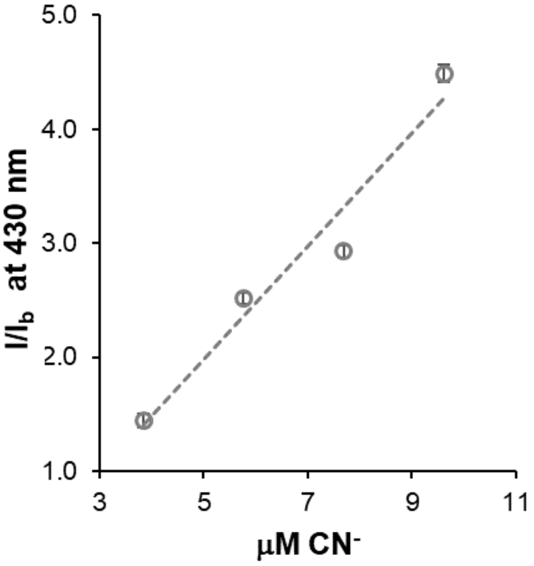

Figure S2. Normalized fluorescence recovery (I/I_b) of a 0.2 μM **1** + 0.1 μM **3** + 2.4 μM Cu^{2+} mixture upon addition of CN^-. Excitation wavelength = 350nm; slope m = 0.5 μM^{-1} CN^-; standard deviation of blank δ = 0.4; analytical detection limit = 3δ/m = 2.5 μM CN^-.

References

1. Fernandes, G. E.; Ugwu, C., Cu^{2+} sensing via noncovalent complexes of fluorescent whitening agents and imidazole-based polymeric dye transfer inhibitors. *J. Appl. Polym. Science* **2020,** 137, 48915.

© 2020 by the authors. Submitted for possible open access publication under the terms and conditions of the Creative Commons Attribution (CC BY) license (http://creativecommons.org/licenses/by/4.0/).
